# On Mixed Types of Fever; in Relation to the Question of the Identity or Non-Identity of the Typhus and Typhoid Poisons

**Published:** 1868-05

**Authors:** Henry Kennedy

**Affiliations:** Dublin


### ON MIXED TYPES OF FEVER; IN RELATION TO THE QUESTION OF THE IDENTITY OR NON-IDENTITY OF THE TYPHUS AND TYPHOID POISONS.

By Dr. HENRY KENNEDY, A.B., Dublin.

[The question of the identity or dissimilarity of typhus and typhoid fevers seems, in England, to be quite decided in favor of the latter. Turn to any work lately published on the subject, or to the doctrines taught at the London hospitals, as shown in the published clinical lectures, and this will forcibly appear. Dr. Kennedy, however, believes the two forms of fever to be identical, both owing their origin to the same poison. He observes that the work of Huss, on this subject, has always been utterly ignored in this country. He saw fevers on a larger scale than any British physician.]

Surely, in such a widespread disease as fever, no one is justified in asserting that what they have seen is what must have been seen by others. We know that even in the type of fever familiar to us as typhus, very great differences exist, and may be constantly seen when the disease attacks several members of the same family. In one, the head symptoms are all in the ascendant; in a second, the chest will be the part attacked; whilst in a third, it will be the stomach, in the form known as gastric fever. Or, again, as regards the spots, the husband will present them and the wife not, or the parents will have them and the children not; or it may be the converse of this. In a family named Bright, of whom eight were in the hospital at the same time, and who were sent in by Dr. Carte, of the Royal Hospital, the children were all spotted, whilst the mother had none, though she had a very severe attack of fever. Again, in three sisters, all adults, who were recently in hospital, only one had the regular spots of the disease; in the other two anything of rash was most indistinct; one of these latter died. Further still, in the great epidemic of 1847–48, the fever was what is known, and had been described previously, as the relapsing fever; that is, it was made up of two parts. There was a sharp attack of fever running on for five or seven days; then a lull of one, two, or three days; and again an onset of fever, usually much severer than the first, and, in very many cases, attended by spots. No one, I think, could have any doubt but that it was one and the same poison that caused the two attacks; and yet, in all the recent and standard works on fever, the relapsing type is described as if it were a totally different fever from typhus, and caused by a different poison. I cannot give in may adhesion to this opinion, for I have as strong a conviction as the nature of the subject admits that the poison of typhus generates not only the type of fever known as relapsing, but other types, such as nervous, gastric, cerebral, etc., as also fever, both without and with spots, and presenting all the variety which they are capable of exhibiting; and if this view of the typhus poison be not held, insuperable difficulties, as it appears to me, must arise when we come to consider analogous diseases to fever, as, for instance, scarlatina. Here, every one must have seen the great variety—I might, also, say contrasts which this disease often presents in the same family and at the same time. Yet no one ever thought of setting down these differences to different poisons; and why it should be necessary as regards fever it is not easy to understand. I must leave this point to others to settle.

From the tenor of these remarks, it will be understood what are the views I hold on the question more immediately under discussion. I believe that the typhus poison is capable of engendering the type of fever known as typhoid or enteric, and that this particular type must be due to some other cause, rather than a specific poison. On the other hand, I hold that the two types can, in the great majority of instances, be distinguished, the one from the other. When I brought the subject first before the Royal Medico-Chirurgical Society of London, in 1860, one of my arguments consisted in the detail of a few cases which were directly opposed to the views of the London physicians. In a later paper, published in the *Dublin Quarterly Journal*, a still larger number of cases were given, and I cannot, I believe, do better now than by giving the briefest sketch of some which have come under my notice within the last two years. But, in truth, I may say the difficulty now consists in selecting the cases, they have become so numerous. So I shall take such as bear most directly on the disputed point.

CASE I. McKeown, farmer, aged 17, having a fine skin, passed through a very severe attack of enteric fever. Every symptom was present, and, during its progress, the brain was much engaged, and the tongue and lips covered with sordes. He made a good though slow recovery.

CASE II. His brother, aged 12, from same room, was admitted under a severe attack of typhus. He had the well-marked and copious rash of the disease, and his face was quite characteristic. He had a sharp attack of diarrhœa, calling for special treatment. My friend Dr. Hudson, was kind enough to come and confirm my diagnosis of this case. It is but right to state that there was an interval of a week between the admission of these two brothers.

These two cases have been given as affording an example of the two types of fever, each well marked, coming from the same room. Others, I know, have met similar examples, and Dr. Croly, of Harcourt Street, has informed me of a very striking one.*

CASE III. McCauley, aged 18, fine skin, was handed over to my care by my friend Dr. Moore. The patient labored under fever, and had the spots of the enteric type very well marked

* After the reading of the paper, Dr. Croly detailed three cases of fever which occurred in the one room. The first was a case of enteric fever, the second a case of typhus, whilst the third was a mixture of the two types.

on abdomen and sides of the thorax, but there was no other sign whatever of this kind of fever. His illness ran on for many days, the chest becoming engaged, and when he left the hospital there were signs about him as if phthisis might supervene.

Cases 4 and 5 were of a similar character to the one just given—that is, with fever, the spots were those of the enteric type, but no other symptom of that kind of fever. As they were published, however, in the *Lancet* for December, 1864, I shall say nothing more of them here.

CASE VI. Podesta, an Italian, 14 years of age, and of a very fine skin, admitted into hospital in September, 1865. In the course of his fever he presented a very good example of the spots said to mark enteric fever. They were few in number, and appeared on the sides of the chest and abdomen. Neither in this case was there any other sign whatever of the enteric type of the disease.

CASE VII. Develin, a young man of 17, admitted into hospital during the present month, April, 1866. He had fever, but not of a severe kind, marked by the usual symptoms, and the tongue red and furred. When he was now six days ill the spots of enteric fever appeared on the chest and abdomen, and in an unusually well-marked form. On the second day of their appearance this patient was seen by the Drs. Martin, from Berlin, who happened to be visiting the hospital. On the third day, however, the number of spots had greatly increased, and become more those of typhus, and, finally, the case, beginning with the spots of enteric fever, became one of irregular typhus.

CASE VIII. Keegan, a man of 27, admitted March, 1865. He was laboring under a heavy spotted fever. Some of the spots were large and dark, some were unusually well defined and red, and disappeared on pressure. The case, however, was one of regular typhus, and the man made a good recovery.

CASE IX. Murphy, girl of 19, whilst passing through a severe attack of fever, with typhus spots, got a very sharp attack of diarrhœa, attended by tympany, and pain on pressing the ilio-cœcal region. Nothing checked this diarrhœa till special treatment was adopted.

CASE X. A dumb girl, aged 24, sent into hospital from the South Union. She had bad fever, being all covered with a copious mealy rash, whilst the tongue, face, and eyes were those which mark typhus. In the progress of the attack she has got severe diarrhœa, attended by tympany, and distinct pain when pressure was made on the ilio-cœcal region, and only here. This complication required specific treatment, and she got steadily but slowly well.

CASE XI. Dixon, a man of 25, of a very fine skin, and thin, admitted into the Cork Street Hospital, laboring under fever, and with a copious rash of typhus spots over him. His general aspect that of the same type of fever. As the disease went on he got severe diarrhœa, the discharges being a light yellow color, and attended by distinct pain in right iliac region, and tympany. This man also required specific treatment, and the attack was one of unusual severity, marked by great distress and restlessness. His recovery, too, was much prolonged by the occurrence of several abscesses.

CASE XII. In February, 1866, Kelly, a man of 19, was admitted into hospital. He was evidently very ill; but the symptoms of typhus and enteric fever were so mixed up that I was quite unable to say to which type of the disease the case ought to be referred. He had a copious rash over the body, and his expression was that of a man in typhus. But he had, also, slight though marked tympany, distinct pain on pressure over the ilio-cœcal region, and a very severe diarrhœa, the discharges being of a light yellow color. He made a very slow recovery. *This man's sister was in hospital at the same time. She had typhus.*

CASE XIII. Woods, a man of 20, came in with a kind of spurious fever on him. He then went out for some days, but returned in a week with every sign of enteric fever on him except the rash. He had, however, spots on him, which to my surprise turned out to be variola in the discrete form. Whilst still in bed from this, he seemed one day to grow suddenly worse, and then typhus in a very severe form declared itself. During all this time he had sharp diarrhœa, and the discharges were those which I believe to be most characteristic of enteric fever, being of a light yellow color. This patient's life was in the balance for many days, but he finally recovered.

CASE XIV. Burn, a girl of 16, admitted in July, 1865. She then labored under a severe attack of typhus, being well spotted. She was so far advanced as to be sent to the Convalescent House, when she again sickened, complaining of her head, and this again followed by great raving and high fever. When now a week ill, the spots of enteric fever made their appearance. These were unusually well marked, being few in number, and confined to the sides of the chest and abdomen; but there was no other symptom whatever of enteric fever, and they were looked for, I need scarcely say, with the greatest minuteness, nor did any such appear. At this stage of her illness, the patient was seen by Dr. Murchison, of London, who was visiting Dublin at the time, but who, I regret to say, I was not fortunate enough to meet.

Such is the series of cases which I wish to bring under the notice of the Association this evening. When added to those already given in the two former papers—and did time permit, I could have given other similar cases—they appear to me to afford the strongest proof the question is capable of eliciting, that we must consider the two types of fever known as typhus and enteric as the result of one poison. If this be not the correct view to take of the matter, I confess myself quite unable to explain the cases of the mixed types detailed this evening; for it must have been observed, as each was given, how the symptoms of each type of fever were mixed up together. As there is not time, however, to go over each symptom in detail, I shall notice but one, on which most, if not all, who hold different views from my own, seemed to have placed the greatest weight of their argument. I mean the spots said to be characteristic of enteric fever. On this point, I think I may say with certainty that these lenticular red spots, and few in number, have not the value which has been given them; for I have seen them now in many instances, and some have been given this evening where, whilst they existed, there was not another symptom of the ileum being engaged—at least I could make out no evidence of such a lesion, though looking specially for it. Here, then, were cases where the particular spots existed, but not the lesion of which they are said to be diagnostic. But, further still, I have given cases to-night where, with the enteric spots, there was also a typhus rash. As bearing on this particular point, I would just recall the case of the man Develin, where the enteric spots first appeared, then the typhus rash, and as this latter disappeared the enteric spots were again visible. If this be not a case in point, I know not what is; and I shall be glad to hear some explanation of this from any gentleman who differs from me. As regards the spots of typhus fever generally, I have got an impression that a good deal of misconception exists. I have heard some speak of the bright and the dark spots, as if there were a difference between them. On this point, I can state with certainty that it is very common to see the two on the same individual, and at the same time. This may be seen on the body itself, but it is more common to have them dark on the body and a bright red on the arms. Again, the spots of enteric fever are described as recurring again and again, and this is quite true. But it does not seem to be so generally known that the same may be seen in typhus, for I have witnessed cases where a distinct second crop of eruption appeared; nor is the observation original, as I have read of it in one of the olden authors, though I cannot at this moment give his name. So also of the statement that petechiæ are never seen on the face. This is positively incorrect, as I have noted several cases where they were quite distinct. But these points are only mentioned here as bearing indirectly on the point under discussion. Still, I think they are enough to show that any positive statements about the rash in fever must be received with caution, as the variety is truly very great. I cannot, however, pursue the subject further here.

In the course of these observations it has been stated that the enteric type of fever must be due to a something else rather than a particular poison; and if asked what that is, I would state my impression that it only occurs in persons of a peculiar constitution, most probably closely connected, if not identical, with the strumous. This idea I have stated before; but every year is increasing my conviction on the point, and if it should turn out to be correct, I need scarcely say how important it would be. I know not whether the idea has struck any one else, but it is not stated in any one of the works on the subject that I have seen. My reasons for holding this view are the following: The enteric fever is very constantly indeed met in persons of a fine skin, and I have now seen several instances where scars, evidently strumous, existed in the neck of persons who had this type of fever. Again, it is much more common under 30 years of age—that is, when the tendency to struma is known to be strongest. I am aware that this remark may be objected to, inasmuch as every type of fever is more frequent under 30; but what I would convey is this, that whilst typhus is common after 30, 40, and 50, enteric fever is exceedingly rare. I myself have not met it in any instance above 35, though it has, I know, been seen later; but, further still, every one is aware that in the course of enteric fever the lungs are very apt to become engaged. But in place of this affection passing off with the fever, as it does in typhus, it is by no means uncommon to meet cases where signs like phthisis declare themselves. The pulse keeps up, sweating occurs, and the cough is very troublesome and hard to relieve. I have said that such is common after enteric fever, and I have been forced to send several out of hospital in this state with the hope that change of air would benefit them, and in some I know that I

heard of subsequently it had proved successful. That the idea I would put forward is not without some surer foundation than mere impression, I may cite the following instance:

Case XV. C., a girl of 16, was admitted into the Cork Street Hospital in January of the present year. She had a very fine skin, with light eyes and hair, and labored under enteric fever in a very well-marked form. The diarrhœa proved most obstinate; but as the abdominal symptoms yielded the lungs got very much engaged from general bronchitis of the minute tubes, and for more than ten days the dyspnœa was of the most urgent character, the lips being quite livid and the distress very great. Though the urgency of this state lessened, the pulse still kept up, and the patient began to have regular sweats, and, finally, I was able to observe that the upper part of the right lung was becoming solidified. Nor did the disease stop here, for in a period of about seven weeks I was able to trace weekly the process of softening going on, till at last a cavity was formed. In this state the patient left the hospital, the physical signs in the top of the lung being those of a cavity, but the rest of the lungs being apparently quite sound, and as the patient's passage had been taken for America, it is just possible the predisposition to tubercle, which seemed so strong in this girl, may be averted, and she might yet live to old age.

Lastly, on this question of the connexion, or supposed connexion, between enteric fever and the strumous diathesis, I would just advert to the great similarity which obtains between the lesion found in the fever and that which so often exists in ordinary phthisis. For my own part, I must say that I have seen many specimens where I could not distinguish them, and I shall be glad to hear any gentleman express his opinion on the point.

The general question brought before the Association this evening is not, as some think, one of mere curiosity. It is of every importance that it should be settled. The diagnosis, prognosis, and treatment of the disease all hinge upon it. For if typhus be the specific fever which some think it, it is obvious that the treatment will differ from what it would do were the enteric lesion present at the same time, and the danger of allowing such a lesion to pursue its course unchecked would indeed be very great. On the other hand, those who hold with myself that the two types of fever may arise from the one poison and coëxist, will always be on the look-out for such a complication, and will act accordingly. For myself, I believe I have often had to deal with such cases, and to alter or modify the treatment as the case required, and that this is not a mere belief I have reserved for this part of my remarks the details of the following cases, which have, however, been on a former occasion detailed:

CASE XVI. A girl 20 years of age was attacked with fever of a severe kind. Raving occurred and petechiæ very early, and these latter spread over the entire body. With these symptoms there was also severe diarrhœa and tympanitis. Matters went from bad to worse, and the patient died about the fourteenth day of the fever. On *post mortem* examination, the lower portion of the ileum was found extensively ulcerated, Peyer's patches being the parts engaged.

CASE XVII. A boy of 14, who had already learned to drink, was attacked with fever. He had much stupor and moaning, both night and day, and he presented a copious petechial rash over the body. With this state he had tympany and diarrhœa, and, finally, involuntary stools and death. On examination, extensive ulceration in patches was found in the lower portion of the ileum. The brain presented the usual appearances found in cases of fever, but in a lesser degree than is common. I should say at the time this case occurred I was much surprised at the result of the *post mortem*, for I then believed the enteric lesion could not exist with regular typhus, which the boy otherwise presented.

CASE XVIII. Hill, a girl of 18, fine skin, was admitted into hospital, after being nine days ill of fever, which presented all the signs of the enteric type, including the spots, which appeared the day after admission. These did not, however, go through the usual course of such spots. They gradually increased in numbers, spreading to the chest, arms, and, finally, to the face, and in this state many of them could not be distinguished from regular petechiæ, being large, dark, and ill-defined. My colleague, as he was then, Dr. Aquilla Smith, saw the patient at this period. By the fourteenth day of the fever, all the signs of enteric fever seemed to have subsided, but there was no corresponding change in the state of the patient. Her nights became restless, she shortly lay on her back, sordes formed on the nostrils, lips, and tongue, and she got great tremor of the upper extremities—in fact, she presented all the signs of well-marked typhus, and died on the twenty-first day of her illness. Except in the lower portion of the ileum, nothing abnormal was found, and here the signs of disease were slight but well marked. Peyer's patches were much plainer than natural, and this became more apparent as the valve was approached, for here one of an inch in length and a-third in breadth was prominent and brought out in strong relief, but it had not ulcerated. The impression given by the inspection was, that irritation had recently been going on in the part but had somewhat subsided. The specimen was exhibited before the Pathological Society.

CASE XIX. Bellew, a servant, aged 45, of tall stature, and thin, admitted in May, 1862, with all the signs of fever in a very severe form. He had to be supported into the hospital, and, though only one week ill, was already densely spotted; his tongue dry and brown; eyes very much injected, and expression heavy. There was also severe diarrhœa, which seemed to cease suddenly within forty-eight hours—that is, about the eighth day of the fever. From this out the attack was as genuine typhus as it is possible to describe. The spots became of the darkest, the mind very confused, with constant rambling, and passing under him. There was difficulty in putting out the tongue, and, late in the illness, hiccup. By the eighteenth day the symptoms had materially improved. The spots were gone, the tongue had expanded and was put out better, and he took support well. It was evident, however, the fever had not resolved itself. The pulse had not fallen in proportion, nor the tongue cleaned, and he still remained heavy and, at times, would ramble. In this state he went on till the twenty-fifth day, when he died. There was no effort at crisis at any time, nor any tympany. I was only able to examine the abdomen. The ileum had no ulceration in it, but it was very red in patches, and the more so the nearer we got to the cœcum. In this last organ, the chief lesion was found, for it was ulcerated in patches, one as large as a shilling. The ascending colon had a number of small and distinct ulcers in it. The glands of the mesentery were not enlarged. It is scarcely necessary to observe that Louis' observations prove that the colon is often engaged in enteric fever, similar to what has been just described.

It appears to me these cases afford as strong a proof as the nature of the subject admits, that the enteric lesion may coëxist with a petechial rash, or, in other words, with typhus fever. On my own mind there now exists not a shadow of doubt of the fact, and if this be not the proper view to take of the matter, I must ask those who differ from me to explain it otherwise. What has been advanced are facts, put what interpretation on them we may. Nor would the slightest difficulty exist in giving other cases, and some striking ones have occurred within the last month; but I prefer now to glance at what others have seen; for if no one else had met similar cases to my own, there would indeed be strong grounds for questioning my powers of observation, and, necessarily, the correctness of what I have stated. I refer, then, my hearers to the lectures of the late Dr. Todd, in which they will find some cases exactly like those given this evening—that is, the enteric type of fever attended by a copious measly rash. Some of these, too, died, and the specific lesion of the intestine was found. Again, in Chambers' "Clinical Lectures," may be found cases of exactly the same kind, and also examples of the two types of fever coming from the same room. Here, then, are two London physicians who fully bear out what has been advanced this evening, and I quote them the more readily, as they have managed to see a class of cases which, by some strange fatality, never seem to have come under the notice of Dr. Jenner and those who agree with him; but, further, I observe that Dr. Lyons, when in the Crimea, met the two types of fever in the combined form, and states, especially in his work, that whilst the rash was genuine typhus, the lesion often found was ulceration of Peyer's patches. In a paper, too, which has just appeared, by Dr. Law, on "Fever," one of the cases given is described as a typhoid case, as I believe it was, and yet the rash was a copious measly one. Lastly, the Drs. Martin, from Berlin, whose names I have already mentioned, told me the two types of fever were commonly looked on as the same disease, and that the enteric type was there called abdominal typhus. I have not the least doubt that had more time been given I could have got further evidence in the same direction; but enough, it appears to me, has been advanced for my present purpose. I do not, for a moment, assert that the question is settled on my side; but I do maintain that enough has been stated this evening to show gentlemen who differ from me the need of a cautious reserve on this question, and in not allowing themselves to come to a decided conclusion till all the facts of the case are clearly before them.

Before concluding these remarks, I would advert for one moment to one other symptom, which some have thought was characteristic of the enteric type of fever—I mean hemorrhage, whether from the nose or the bowels. The London physicians especially, look on it in this light, but it certainly is not correct as regards Dublin. With us, typhus often exhibits epistaxis, both in its earlier and more advanced stages. In the summer it is very common, particularly when the temperature ranges high; but it is much more frequent in some years than others. And, again, as regards bleeding from the intestines, I myself have put on record some thirty cases—most of them regular typhus—in which bleedings, more or less severe, occurred, and in some that proved fatal and were examined not a trace of ulceration was found. So that bleedings cannot in any way be considered as specially diagnostic of enteric fever, and I do believe the same may be said of any other symptom that might be chosen. I would repeat, however, that it is quite another matter distinguishing between the several types of fever. This can very usually be done, and ought, of course, always be attempted; but that the types of fever will often be found united I cannot doubt, and I think the time will come when the natural history of fevers—for this is really the question at issue—will be looked on in a very different light from what it at present is.

On the treatment of fever, I have here little to say. As a single remedy, and in the ordinary typhus, I find balm still the best. It seems to me to act as an antiseptic, and to fulfil the indications required better than any other agent with which I am acquainted. I consider, too, that, to a certain extent, it supplies the place of wine: and this is no little matter to be able to say of it. Under its use the mortality, in spotted cases, has, I believe, been reduced to the lowest on record. But having spoken of these several points on a former occasion, as likewise the dose and method of using it, and the precautions to be adopted, I shall not enter upon them further now.

Of the treatment of the enteric type of fever, I have only to repeat that, when seen early, it appears to me the most amenable of the several forms of the disease. I mention this, because elsewhere, particularly in London, it seems to be a very fatal disease. Like typhus, it appears as if it were a more severe disease there than with us in Dublin. Though not easily accounted for, this may be so. Still, my conviction is, that treatment has a more decided effect on it than any other type of fever. For myself, I use astringents, and from an early stage of the attack, and it is the dilute sulphuric acid on which I chiefly rely. This is the medicine recommended by Huss, and in the proportion of one, two, or three drachms to an eight-ounce mixture, I have found it most useful. Two or three drops of laudanum are added to each ounce of the mixture, which is repeated according to the urgency of the case. It is, however, to be observed that the diarrhœa is only to be moderated, not directly checked; and this rule is the more important the earlier the disease is seen. If the diarrhœa be stopped too soon or too suddenly, mischief elsewhere than in the intestines will arise. It may be in the chest, or the brain may be the organ that suffers. Several such cases have come under my notice; but though some of these were severe, none proved fatal. One, however, was so remarkable that I must give it here; for the checking the diarrhœa had, or seemed to have, the effect of altering the type of fever under my very eye:

Case XX. Kelly, a man of 19, having a fine skin, was admitted into hospital, laboring under the enteric type of fever in a well-marked form. He presented the characteristic diarrhœa and also the spots, and had been nine days ill. After three doses of the acid mixture the diarrhœa suddenly ceased, and was at once succeeded by symptoms referred to the head. His eyes, which before had been quite clear, became deeply injected; he complained of headache, his face flushed, he began to rave, and in the course of two days he presented the countenance of a well-marked typhus case, his tongue and lips being then covered with sordes. In this state, and when now about twelve days ill, his nose began to bleed, and this was repeated daily three times, so that he bled in all on four occasions. The first of these the bleeding was much the most, and they were all so obviously beneficial that they were not interfered with. The patient made a good though very slow recovery. There was no recurrence of symptoms referable to the intestines. I have seen several instances like the one just given, but none so striking, and none which proved fatal. When, however, any similar instance occurs, it may be assumed that the case is quite within our control.—*Medical Press and Circular, June 20, 1866, p. 647.*

